# Endothelial dysfunction in the pathogenesis of pre-eclampsia in Ghanaian women

**DOI:** 10.1186/s12899-017-0029-4

**Published:** 2017-03-29

**Authors:** Kwame Adu-Bonsaffoh, Daniel Ansong Antwi, Ben Gyan, Samuel Amenyi Obed

**Affiliations:** 1 Department of Obstetrics and Gynecology, Korle Bu Teaching Hospital, Accra, Ghana; 2 Department of Physiology, School of Biomedical and Allied Sciences, College of Health Sciences, University of Ghana, Accra, Ghana; 3 Department of Obstetrics and Gynecology, School of Medicine and Dentistry, College of Health Sciences, University of Ghana, Accra, Ghana; 4 Department of Immunology, Noguchi Memorial Institute for Medical Research, University of Ghana, Accra, Ghana

**Keywords:** Endothelial dysfunction, Pre-eclampsia, Vascular endothelial growth factor, Ghana

## Abstract

**Background:**

Pre-eclampsia (PE) remains a disease of theories despite extensive research into its etiology. Alteration in the production of vascular endothelial growth factor (VEGF), a biomarker of endothelial dysfunction, is associated with pre-eclampsia although conflicting reports have been reported. The aim of the study was to determine and compare maternal serum levels of VEGF among pre-eclamptics, normotensive non pregnant and pregnant women.

This was a cross-sectional study involving 100 women with pre-eclampsia, 102 women with normotensive pregnancy and 75 normotensives who were not pregnant. The study was carried out at Korle Bu Teaching Hospital (KBTH) from April to June in 2011. Basic socio-demographic and obstetric data were obtained by means of structured questionnaire. Following venesection, about 5mls of blood was sampled from the participants for the various tests. Enzyme Linked Immunosorbent Assay was used to determine the maternal serum levels of free VEGF. Data analysis was performed using SPSS version 20.

**Results:**

Significant reduction in median serum levels of free VEGF was seen in both, normal pregnant [84.06 pg/ml (IQR: 78.90–99.67)] and pre-eclamptic women [4.71 pg/ml, (IQR: 3.41–7.93)] compared to the non-pregnant (395.85 pg/ml, IQR 234.93–625) with *p* < 0.001; the reduction was far greater in the pre-eclamptic group compared to that of normotensive pregnant group (*p* < 0.001). Early-onset pre-eclampsia had significantly more severe reduction in free VEGF levels (3.89, IQR: 2.60–5.67 pg/ml) compared to that of late onset PE (5.23, IQR: 3.78–16.97 pg/ml) with *p*<0.001 indicating a severer endothelial damage in former.

**Conclusions:**

Endothelial dysfunction contributes significantly to the pathogenesis of pre-eclampsia as demonstrated by profound decrease in maternal serum VEGF levels in PE compared to normotensive pregnancy and non-pregnancy state. The pathophysiology of early-onset pre-eclampsia may be partly explained by marked reduction in free serum VEGF levels with resultant severe endothelial dysfunction.

## Background

Pre-eclampsia (PE) is a multi-system pregnancy specific disorder, characterized by new onset of hypertension and proteinuria developing after the gestational age of 20 weeks [1–3]. It remains a major cause of both maternal and perinatal mortality and morbidity [1–4]. Maternal mortality in PE is mostly due to major complications such as eclampsia, cerebral hemorrhage and renal failure [3, 4]. Currently, hypertensive disorders including pre-eclampsia are the commonest cause of maternal demise at the Korle Bu Teaching Hospital (KBTH) where the current study was carried out [5].

Pre-eclampsia remains a disease of theories despite extensive research into its etiology as the exact cause remains uncertain [1, 2]. Dysfunctional endothelium is hypothesized to contribute significantly in the pathogenesis of pre-eclampsia. Alteration in the production of VEGF, a biomarker of endothelial dysfunction, has been associated with pre-eclampsia although conflicting of increased, decrease and normal maternal serum VEGF levels have been reported [6, 7]. Adequate knowledge about the pathophysiologic role of VEGF in pre-eclampsia is vital in devising appropriate screening tests to facilitate early detection and treatment of the disease.

Pre-eclampsia is hypothesized to be associated with alterations in maternal plasma concentrations of vascular endothelial growth factor (VEGF), and other related growth factors and their receptors. VEGF has been implicated in pre-eclampsia because it contributes significantly to physiological vasculogenesis and vascular permeability [7]. VEGF is known to stimulate the production of nitric oxide which is a potent vasodilator associated with gestational vasodilation characteristic of normal pregnancies. In addition, there is genetic evidence from VEGF conditional knockout mice that interference with VEGF signaling in the kidneys leads to clinical features of PE such as proteinuria and glomerular endotheliosis [8]. The major important attributes of VEGF include stimulation of angiogenesis and promotion vasodilation by stimulating the production of nitric oxide and prostacyclin which are important signaling molecules [7, 8].

In Ghana and most parts of Africa, there is limited data regarding the contribution of VEGF to the pathophysiology of PE indicating a significant gap in knowledge in our indigenous setting. This study aimed at determining whether or not endothelial dysfunction has a significant contribution in the pathogenesis of pre-eclampsia by comparing maternal serum levels of free VEGF in normotensive non-pregnant, normotensive pregnant and pre-eclamptic women.

## Methods

This was a cross-sectional study conducted at the Department of Obstetrics and Gynecology of the KBTH in Accra from April to June, 2011. Korle Bu Teaching Hospital is a tertiary centre and the biggest teaching hospital in Ghana with a total bed capacity of 1600 with about 12,000 deliveries per year. It is a training institution for the College of Health Sciences of the University of Ghana. Antenatal care is offered on a daily basis from Monday to Friday at KBTH and pregnant women are usually seen after every four weeks until 28 weeks, fortnightly until 36 weeks and then weekly till delivery.

The study protocol was approved by the Ethical and Protocol Review Committee of the School of Medicine and Dentistry, of the University of Ghana. All the study participants provided informed consent. Study participants who could not give informed consent were excluded from the study and the informed consent was not obtained from next of kins, caretakers or guardians. In this study, pre-eclampsia was defined as new onset of hypertension (systolic blood pressure of 140 mm Hg or more and/or diastolic blood pressure of 90 mm Hg or more) and proteinuria after the gestational age of 20 weeks [3]. Early- and late-onset pre-eclampsia were defined as PE that develops before and at or after gestational age of 34 weeks respectively [9, 10]. Mild and severe pre-eclampsia were defined as maternal systolic blood pressure of 160 mm Hg or more and/or diastolic blood pressure of 110 mm Hg or more on two occasions at least 6 h apart [3]. Proteinuria was determined using the semi-quantitative dipstick testing and significant proteinuria was defined as the presence of urine proteins of 1+ or more corresponding to 0.3 g or more of protein in a 24-h urine specimen [3].

The detailed methodology has been published elsewhere [11]. Briefly, the study participants were recruited using systematic random sampling. Structured questionnaire was used to collect data on basic demographic and obstetric characteristics of the study participants (Table 1). Other necessary data about the participants such as blood pressure at diagnosis and delivery, birth weights of the babies and gestational ages at delivery were retrieved from the medical records. The study participants were given identification numbers recorded in a log book which was kept securely during the study period. All the data obtained were also kept securely and the study participants were identified only by the study identification number and not by their names.

**Table 1:** Demographic and clinical characteristics of study participants

Variable	Normotensive Non pregnant women n (75)	Normotensive pregnant women n (102)	Pre-eclamptic women n (100)	Total N (277)
Age (years)				
<20	1 (1.3)	5 (4.9)	2 (2.0)	8 (2.9)
20–34	51 (68.0)	85 (83.3)	69 (69.0)	205 (74.0)
≥35	23 (30.7)	12 (11.8)	29 (29.0)	64 (23.1)
Parity				
0	38 (50.7)	35 (34.3)	61 (61.0)	134 (48.4)
1	21 (28.0)	34 (33.3)	20 (20.0)	75 (27.1)
2≥	16 (21.3)	33 (32.4)	19 (19.0)	68 (24.5)
GA at delivery				
<34	-	3 (0.3)	10 (10.0)	13 (6.4)
34–36	-	5 (0.5)	44 (44.0)	49 (24.3)
≥37	-	94 (92.2)	46 (46.0)	140 (69.3)

*GA* Gestational age in weeks

### Blood sampling

Venous blood samples were taken from the antecubital fossae of the study participant for study purposes after application of a tourniquet. Blood samples were obtained upon presentation from all patients and controls. In pre-eclamptic patients, the blood samples were obtained at the time of diagnosis prior to the commencement of any antihypertensive treatment including the use of magnesium sulphate. In the normotensive non pregnant women, the blood samples were collected in during the follicular phase of the menstrual cycle and were uniform for all such participants. Non pregnant women who had chronic medical conditions such as hypertension, sickle cell disease, asthma, cardiac disease and those on any hormonal medications such as contraceptives were excluded from the study. The blood samples were collected into plain sterile test tubes and no inhibitors were added to samples at the time of collection. The blood sample was centrifuged for 15 min at 1000 × g within one hour of collection and the serum was stored in a fridge at −80 °C before the laboratory assays were carried out. Repeated freeze-thaw cycles were avoided.

### Measurement of VEGF levels

Human VEGF ELISA test kit (Quantikine, USA) was used to determine serum levels of VEGF. The VEGF assay was carried out strictly according to the instructions of the manufacturers. Recombinant human VEGF standard in a 2-fold serial dilution from 3000 pg/ml to 31.25 pg/ml using 1.0 mL of calibrator diluent RD6U was employed. 100uL of standard, control and serum sample were added to the wells and incubated for 2 hour at room temperature. After a 4 times washing step, 200 μL of VEGF Conjugate (polyclonal antibody against VEGF conjugated to horseradish peroxidase) was added to each well and the plates were incubated for 2 hour at room temperature. The plates were then washed 5 times and developed with 200 μL/well of 3,3′,5,5′-Tetramethylbenzidine TMB (4390A, Kem-En-Tec Diagnostics, USA) substrate for 25 min. The reaction was stopped using a sulfuric acid stop solution and optical densities read at 450 nm. The mean absorbance for each set of duplicate standards, controls and samples were then calculated, and the average zero standard optical density was subtracted to obtain the required values.

### Statistical analyses

Data analysis was performed with SPSS version 20 (Microsoft company, USA). Descriptive analysis was performed and the results were presented in percentages (for categorical data), mean ± standard deviation (for continuous data). The median serum levels of VEGF between the normotensive non-pregnant, normotensive pregnant and pre-eclamptic women were also compared using non-parametric Kruskal-Wallis test and results presented using box plots. Post hoc analysis was then performed using the Bonferroni’s test to determine the specific differences following the Kruskal-Wallis test. The pre-eclamptic group was, further, sub-classified into early- and late-onset pre-eclampsia. The maternal age, systolic and diastolic blood pressure at diagnosis and delivery and birth weight were compared between the two groups using independent student *t*-test and the results were presented in means ± standard deviation. Median serum levels of VEGF between early and late –onset pre-eclamptic women were also compared using non-parametric Mann–Whitney test and results presented using box plots. Also, the median serum levels of VEGF between mild and severe pre-eclamptic based on diastolic, systolic blood pressures or both compared using non-parametric Mann–Whitney test. A *p*-value of less than 0.05 was interpreted as statistically significant at 95% confidence interval.

## Results

The study included 100 women with pre-eclampsia, 102 normotensive pregnant (control group 1) and 75 normotensive women who were not pregnant (control group 2) resulting in a total of 277 participants. Table 1 indicates the characteristics of the study participants. Significant differences were found between the pre-eclamptic and normotensive pregnant women in terms of the mean systolic and diastolic at the time of recruitement (*p*<0.001).

**Fig. 1:**
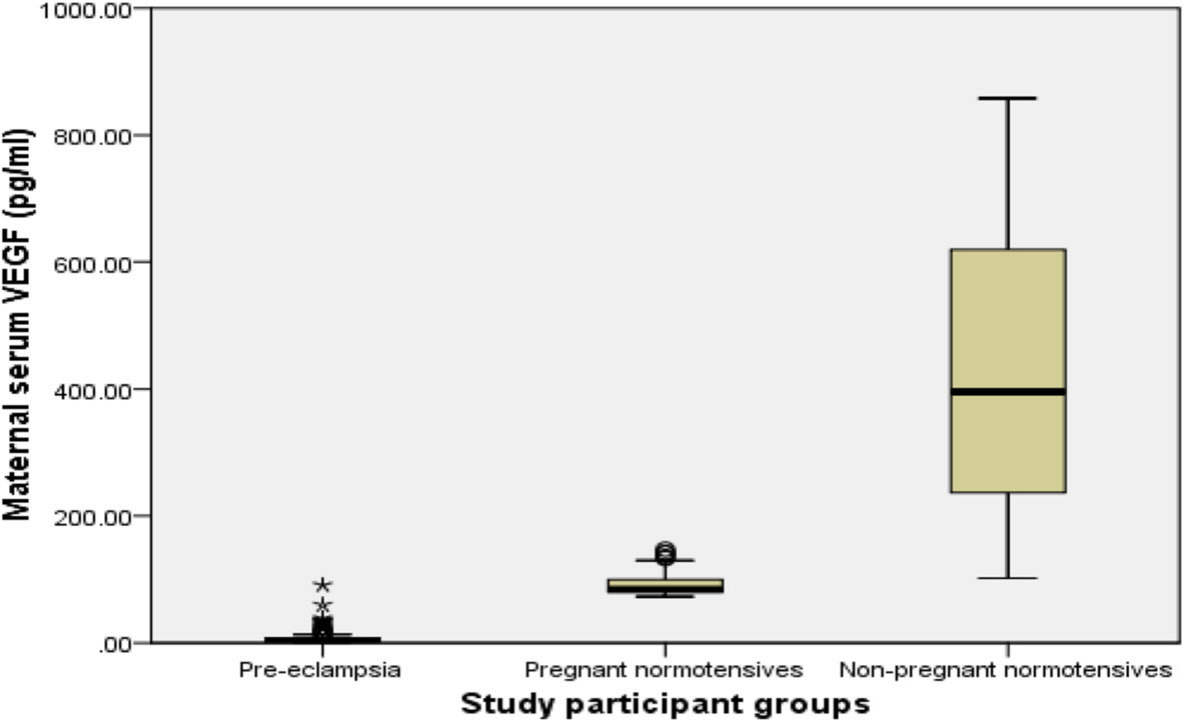
Maternal serum levels of free VEGF in pre-eclampsia, normotensive pregnancy, and non-pregnant state

**Fig. 2:**
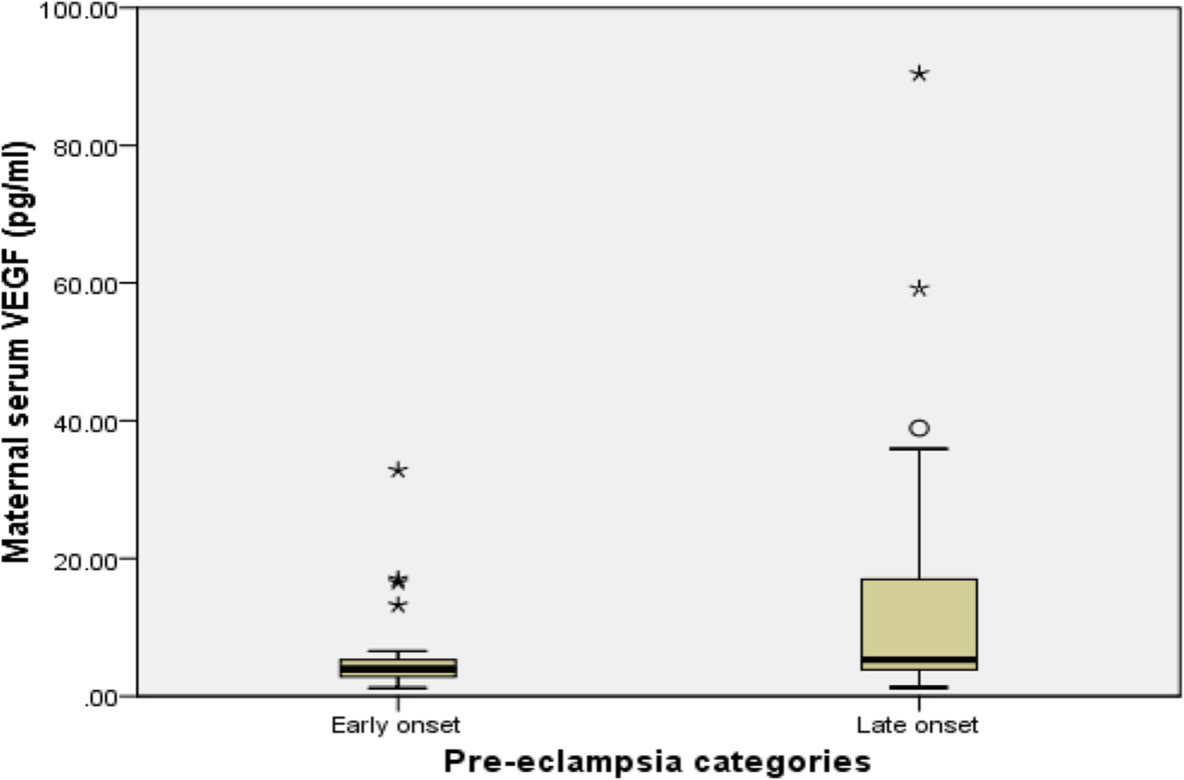
Maternal serum levels of free VEGF in early- and late-onset pre-eclampsia

Most (74%) of the study participants were between 20 to 34 age group. Regarding parity distribution, 61%, 20% and 19% of the pre-eclamptics were nulliparous, primiparous and multiparous respectively. At the time of diagnosis, 35 (35%), 44 (44%) and 21 (21%) of the pre-eclamptics had gestational ages of <34 weeks, between 34 to 36 and ≥37 weeks respectively. There were 35 (35%) and 65 (65%) women with early- and late onset PE respectively. No statistically significant differences were found with respect to age, parity between the two groups. There were significant differences between early- and late onset PE with respect to birth weight, gestational age at diagnosis and delivery, blood pressures at diagnosis and delivery (Table 2). Also, significant differences were determined between the median serum VEGF levels in normotensive non-pregnant, pre-eclamptic and normotensive pregnant women with *p* < 0.001. The median serum VEGF levels in non-pregnant, pre-eclamptic and normal pregnant women are 395.85 pg/ml (IQR: 234.93–625), 4.71 pg/ml (IQR: 3.41–7.93) and 84.06 pg/ml (IQR: 78.90–99.67) respectively (*p* < 0.001) (Fig. 1). Posthoc analysis using the Mann Whitney test showed significant differences in the median serum VEGF levels between pre-eclampsia and normotensive pregnancy (*p* < 0.001), eclampsia and normotensive pregnancy (*p* < 0.001) and normotensive pregnancy and non-pregnancy states (*p* < 0.001). Maternal serum median VEGF levels were significantly lower in early-onset PE (3.89, IQR 2.60–5.67 pg/ml) compared to that of late-onset disease (5.23, IQR 3.78–16.97 pg/ml) with *p*<0.001 (Fig. 2). There was a significant difference in birth weight of the neonates (in grams) between the normotensive pregnant (3197.06 ± 538.92) and pre-eclamptic women (2422.10 ± 716.76) with a p-value less than 0.001.

**Table 2:** Demographic and clinical characteristics women with early- and late-onset pre-eclampsia

Variable	Early onset PE	Late onset PE	*P*-value
	*N* = 35	*N* = 65	
Age (years)	30.66 ± 5.75	29.09 ± 5.30	0.175
GA at diagnosis (weeks)	30.74 ± 2.12	35.77 ± 1.49	<0.001^b^
Ga at Delivery (weeks)	34.29 ± 2.28	36.80 ± 1.20	<0.001^b^
SBP at diagnosis (mmHg)	175.43 ± 28.84	160.42 ± 12.58	<0.001^b^
DBP at diagnosis (mmHg)	110.86 ± 12.46	103.82 ± 10.58	0.004^a^
Birth weight (g)	1862.86 ± 665.57	2723.23 ± 544.26	<0.001^b^
SBP at delivery (mmHg)	186.97 ± 17.32	174.15 ± 14.88	<0.001^b^
DBP at delivery (mmHg)	115.09 ± 9.80	110.00 ± 9.52	0.013^a^

*DBP* Diastolic blood pressure, *SBP* Systolic blood pressure, *PE* Pre-eclampsia, *GA* Gestational age

^a^Significant; ^b^very Significant; All the values in the Table are presented in mean ± SD

**Fig. 3:**
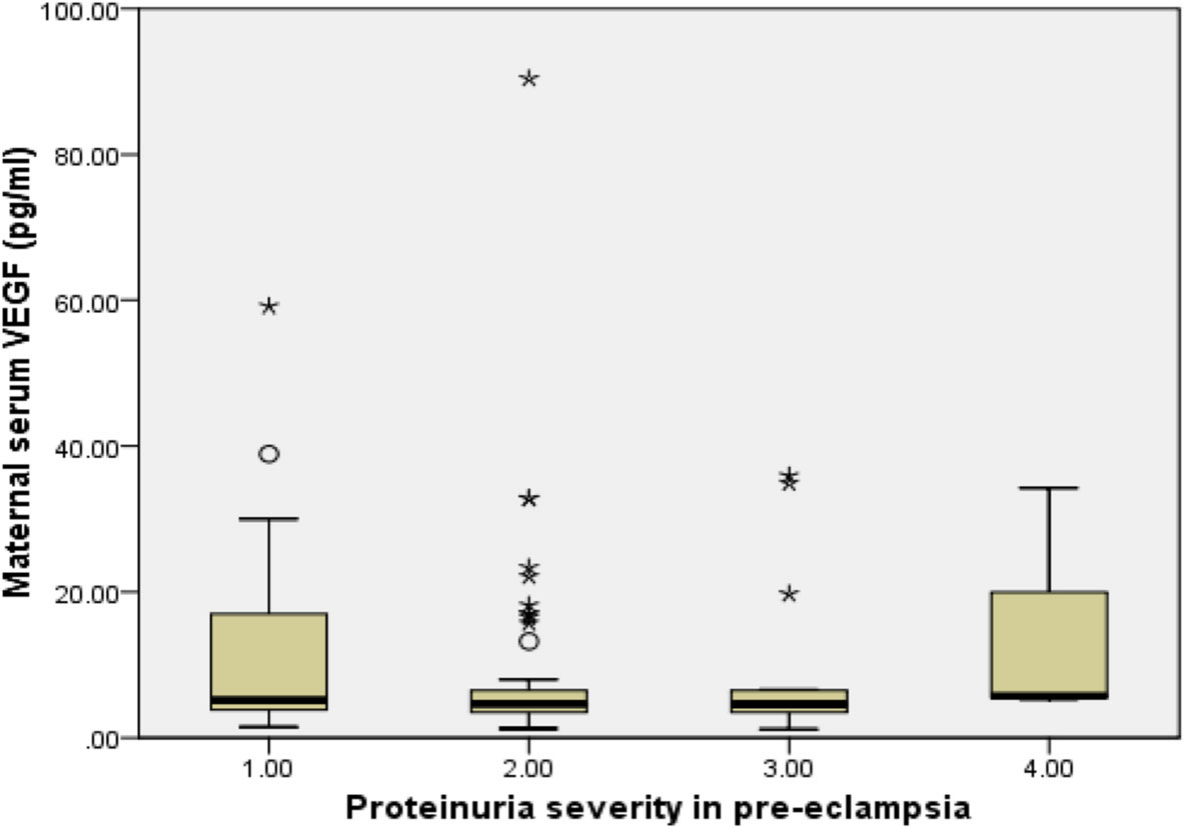
Maternal serum VEGF levels and severity of proteinuria in pre-eclampsia

Mild and severe pre-eclampsia occurred in 47% and 53% of pre-eclamptics respectively when classified using diastolic pressure only, 37% and 63% respectively when classified using systolic pressure only, and 24% and 76% respectively when classified based on both diastolic and systolic pressures combined Table 3. There were no significant differences across the various categories regarding mean gestational age at diagnosis and delivery. However, there were significant differences between the two disease entities across the various categories of classification with respect to the systolic and diastolic blood pressures. Generally, there were no significant differences between severe and mild disease across the various categorizations with respect to the maternal serum median levels of vascular endothelial growth factor (Table 3). Among the pre-eclamptics, proteinuria occurred in 26 (26.0%), 54 (54.0%), 17 (17.0%) and 3 (3.0%) women in severity order of +1, +2, +3 and +4 respectively. There was no statistically significant difference (*p* = 0.281) between the maternal serum VEGF levels and the severity of proteinuria in women with pre-eclampsia (Fig. 3). Among women with early and late onset PE, +1 proteinuria occurred in 8 (22.9%) and 18 (27.7%), +2 proteinuria occurred in 20 (57.1%) and 34 (52.3%), +3 proteinuria in 6 (17.1%) and 11 (16.9%), +4 proteinuria in 1 (2.9%) and 2 (1.5%) women respectively.

**Table 3:** Characteristics of women with mild and severe pre-eclampsia at Korle Bu Teaching Hospital

	Diastolic Blood Pressure classification			Systolic Blood Pressure classification			Diastolic and Systolic Blood Pressure classification		
Variable	Mild PE	Severe PE	*P* -value	Mild PE	Severe PE	*P* -value	Mild PE	Severe PE	*P* -value
	*N* = 47	*N* = 53		*N* = 37	*N* = 63		*N* = 24	*N* = 76	
Age (years)	29.68 ± 5.32	29.60 ± 5.67	0.944	30.73 ± 5.39	29.54 ± 5.47	0.128	31.75 ± 4.55	28.97 ± 5.61	0.030
DBP (mmHg)	96.77 ± 5.92	114.72 ± 8.68	<0.001	100.76 ± 8.0	109.52 ± 12.37	<0.001	95.75 ± 4.98	109.61 ± 11.25	<0.001
SBP (mmHg)	156.74 ± 12.02	173.58 ± 23.95	<0.001	147.49 ± 5.40	176.35 ± 19.29	<0.001	146.96 ± 6.13	171.58 ± 20.53	<0.001
GA1	34.43 ± 2.93	33.64 ± 2.96	0.187	33.78 ± 3.21	34.14 ± 2.82	0.561	33.54 ± 3.48	34.16 ± 2.79	0.377
GA 2	36.32 ± 1.64	35.57 ± 2.30	0.066	35.95 ± 1.81	35.90 ± 2.18	0.923	36.08 ± 1.95	35.87 ± 2.08	0.656
Birth weight (g)	2576.81 ± 669.76	2284.91 ± 735.15	0.041	2370.54 ± 683.78	2452.38 ± 739.14	0.584	2387.92 ± 751.83	2432.89 ± 710.14	0.790
Median VEGF (pg/ml)	4.84 (3.44–16.97)	4.60 (3.40–6.48)	0.227	4.68 (3.40–6.70)	4.73 (3.77–13.18)	0.689	2.68 (2.98–4.64)	4.71 (3.70–7.93)	0.654

*PE* pre-eclampsia, *GA1* gestational age at diagnosis, *GA2* gestational age at delivery, *DBP* diastolic blood pressure at diagnosis, *SBP* systolic blood pressure at diagnosis

## Discussion

The contribution of vascular endothelial growth factor (VEGF) in pathophysiology of pre-eclampsia has received huge attention recently although conflicting reports has been reported in previous studies [6, 7, 12]. This study has demonstrated a significant reduction in maternal serum free VEGF levels in both normotensive pregnancy and pre-eclampsia compared to the normotensive non-pregnant state. However, the reduction in maternal serum level of VEGF in pre-eclampsia was significantly far greater than that of the normal pregnancy (*p*<0.001). This shows that although there was significant decrease in serum VEGF levels in normal pregnancy as well, it did not probably reach the critical threshold that could cause significant endothelial dysfunction necessary to result in clinical evidence of pre-eclampsia.

The results obtained in our study agree with other reports that have demonstrated severe reductions in maternal serum VEGF levels in PE [13–15]. The marked reduction in serum VEGF levels determined in association with pre-eclampsia could be attributed to high soluble fms-like tyrosine kinase-1 (sflt-1) levels. Soluble flt-1 usually binds to and inhibits multiple pro-angiogenic proteins such as VEGF and placental growth factor (PIGF) by preventing their communication with the respective endothelial cell receptors [16]. The physiological effects of VEGF are mediated via its binding to two high-affinity receptor tyrosine kinases which are selectively expressed on the surface of the vascular endothelial cell [6, 12, 13]. Soluble flt-1 is a splice variant of flt-1 having the extracellular domain but lacking both the transmembrane and cytoplasmic domains which were excised post-transcriptionally. Soluble flt-1 acts as a potent antagonist by binding to VEGF and inhibits its biologic activity [16].

In this study, maternal serum free VEGF levels were found to be markedly lower in the early compared to the late onset PE (*p*<0.001) buttressing the fact that early onset disease is a severer disease leading to a more pronounced endothelial dysfunction. In severe pre-eclampsia, placental cells secrete high levels of soluble isoform of flt-1 (an antiangiogenic factor) which interacts and neutralizes the effects of VEGF and PIGF [9]. The finding of markedly reduced levels of free VEGF in early onset pre-eclampsia is consistent with other reports and this suggests marked endothelial dysfunction and dysregulation manifesting as severe hypertension and proteinuria. This is evidenced by the significantly high blood pressures recorded in early compared to late onset PE. The higher the serum sflt-1 levels, the lower the bioavailable VEGF levels and the severer the endothelial dysfunction [17] resulting in severer clinical manifestation. The markedly reduced free VEGF levels determined in early onset PE points to the possibility of different pathophysiological mechanisms and clinical presentations.

We did not measure maternal serum levels of sflt-1 in association with VEGF, however, there is enough evidence from previous studies demonstrating that sflt-1 levels are significantly elevated in pre-eclampsia causing marked reduction in the circulating levels of free VEGF [13, 15, 17]. Increased soluble flt-1 levels produces hypertension, proteinuria and glomerular endotheliosis in both pregnant and non-pregnant rats and these features also characterize pre-eclampsia [18]. This strongly suggests that significant decrease in free VEGF levels resulting in endothelial dysfunction is implicated in the pathogenesis of pre-eclampsia. However, other studies have demonstrated that maternal serum free VEGF levels may be increased, normal or decreased in uncomplicated pregnancy [12, 19, 20]. The fact that maternal serum levels of sflt-1 were not measured concurrently with VEGF is considered a limitation of this study since it would have provided more insight into the relativity of these factors to endothelial damage in pre-eclampsia.

However, there were no significant differences between severe and mild disease with respect to the median maternal vascular endothelial growth factor levels when pre-eclampsia was categorized based on diastolic, systolic blood pressure values or both (Table 3). These findings might partly be due to the fact that clinical disease severity based on the blood pressure readings alone might not correlate well with the severity of endothelial functional and morphological injury in pre-eclampsia. Therefore, clinical decision making based on the severity of the blood pressure alone, especially regarding termination of pregnancy might not always be justifiable. This buttresses the need for biochemical monitoring alongside clinical features in the management of pre-eclampsia. However, there was a tendency of significantly lower VEGF levels in severe compared when pre-eclampsia categorized based on diastolic blood pressure alone although the difference did not reach statistical significance.

The conflicting results as demonstrated by other researchers with respect to maternal serum VEGF levels in normotensive pregnancy and pre-eclampsia could probably be attributed to different gestational ages at which the women were studied [19] as well as differences in methodology. In this study, all the gravid women were studied at or after the gestational age of 28 weeks and therefore there is limited confounding effect that could have been introduced by differences in gestational age. The markedly reduced serum VEGF levels in pre-eclampsia are not unexpected because hypertension and proteinuria are the main signs of VEGF deficiency.

Maynard and colleagues determined that administration of exogenous VEGF therapy might restore normal endothelial function in pre-eclampsia [7]. In this regard, therapy using pro-angiogenic analogues might be relevant in the management of pre-eclampsia by improving vascular endothelial function. The use of nicotine may be clinically beneficial in the treatment of pre-eclampsia since it is associated with stimulation of angiogenesis [14] but this is not recommended in pre-eclampsia as it is associated with intrauterine growth restriction which is also a recognized complication of pre-eclampsia. However, it has been suggested that short term use of nicotine in the management of severe pre-eclampsia might be helpful since it is associated with reduction in sFlt1 levels in humans [7]. Moreover, lower incidence of pre-eclampsia has been shown in individuals who smoke due to reduced serum sflt-1 [21].

Intrauterine growth restriction (IUGR) is a recognized complication of pre-eclampsia and the two conditions share many clinical and pathologic features [7]. Alteration in angiogenic factors in IUGR without pre-eclampsia is less pronounced than in pre-eclampsia. Although IUGR is frequently associated with early onset PE it can also occur without pre-eclampsia [7]. The pathophysiological mechanisms of these two related conditions are associated with the failure of the trophoblasts to achieve deeper invasion of the maternal spiral arteries during early pregnancy [22]. The mean birth weight and gestational age at delivery determined in our study were significantly lower among women with pre-eclampsia compared to normotensive pregnancy women (Table 1). Similarly, early onset PE delivered at an earlier gestational age with significantly smaller mean birth weight compared late onset disease (Table 2). Fetal growth restriction is a major complication of pre-eclampsia [3, 7] and it might be responsible for the lower birth weights recorded in PE and especially in the early onset type.

However, significant elevation of VEGF levels in pre-eclampsia has also been reported [20]. The explanation for the increase in VEGF was its association with extravasation of plasma proteins and proteinuria. This particular characteristic of VEGF is related to its ability to regulate endothelial cell function and vascular permeability at microcirculation level [20].

The strength of this study revolves around the large numbers of participants involved and the fact that it is the first study in Ghana relating the pathophysiology of pre-eclampsia to VEGF. However, the study has a number of limitations and this include the determination of VEGF in serum rather than plasma which is more reliable. There is evidence that platelets and leukocytes release VEGF during blood clotting associated with the use of serum [23] and this might have influenced the result obtained. Also, specific inhibitors could prevent the release of VEGF from platelets and leukocytes in the serum [23] but none of such inhibitors were added to the samples. Another limitation is associated with the use of semi-quantitative method in the determination of proteinuria compared with the 24 h urine collection method which is more reproducible.

## Conclusions

This study has demonstrated marked reduction in maternal serum bioavailable VEGF levels in pre-eclampsia compared to normotensive pregnancy suggesting marked vascular endothelial cell dysfunction resulting in generalized vasoconstriction, hypertension and proteinuria. This might be associated with the lower birth weight recorded in the pre-eclamptic group as a part of intrauterine growth restriction which commonly complicates pre-eclampsia. The reduction in serum free VEGF levels was markedly more severe in early-onset pre-eclampsia coupled with severe low birth weight compared to late onset disease indicating a severer endothelial injury in the former.

## Abbreviations

ANOVA: Analysis of variance
DBP: Diastolic blood pressure
ELISA: Enzyme linked immunosorbent assay
GA: Gestational age
HELLP: Hemolysis, elevated liver enzymes, low platelets
IUGR: Intrauterine growth restriction
KBTH: Korle Bu Teaching Hospital
PE: Pre-eclampsia
PIGF: Placental growth factor
SBP: Systolic blood pressure
VEGF: Vascular endothelial dysfunction.
